# Duplex Shiny app quantification of the sepsis biomarkers C-reactive protein and interleukin-6 in a fast quantum dot labeled lateral flow assay

**DOI:** 10.1186/s12951-020-00688-1

**Published:** 2020-09-10

**Authors:** Christoph Ruppert, Lars Kaiser, Lisa Johanna Jacob, Stefan Laufer, Matthias Kohl, Hans-Peter Deigner

**Affiliations:** 1 Medical and Life Sciences Faculty, Furtwangen University, Jakob-Kienzle Str. 17, 78054 Villingen-Schwenningen, Germany; 2 Institute of Precision Medicine, Furtwangen University, Jakob-Kienzle Str. 17, 78054 Villingen-Schwenningen, Germany; 3 Department of Pharmaceutical Chemistry, Pharmaceutical Institute, University of Tuebingen, Auf der Morgenstelle 8, 72076 Tübingen, Germany; 4 Institute of Pharmaceutical Sciences, University of Freiburg, Albertstraße 25, 79104 Freiburg, Germany; 5 EXIM Department, Fraunhofer Institute IZI, Leipzig, Schillingallee 68, 18057 Rostock, Germany; 6 Faculty of Science, Tuebingen University, Auf der Morgenstelle 8, 72076 Tübingen, Germany

**Keywords:** Duplex lateral flow assay, Point-of-care diagnostics, Nanoparticles, Quantum dots, Image processing, R-package, Shiny app, Sandwich immunoassay, Multiplexing, Conjugation chemistry

## Abstract

Fast point-of-care (POC) diagnostics represent an unmet medical need and include applications such as lateral flow assays (LFAs) for the diagnosis of sepsis and consequences of cytokine storms and for the treatment of COVID-19 and other systemic, inflammatory events not caused by infection. Because of the complex pathophysiology of sepsis, multiple biomarkers must be analyzed to compensate for the low sensitivity and specificity of single biomarker targets. Conventional LFAs, such as gold nanoparticle dyed assays, are limited to approximately five targets—the maximum number of test lines on an assay. To increase the information obtainable from each test line, we combined green and red emitting quantum dots (QDs) as labels for C-reactive protein (CRP) and interleukin-6 (IL-6) antibodies in an optical duplex immunoassay. CdSe-QDs with sharp and tunable emission bands were used to simultaneously quantify CRP and IL-6 in a single test line, by using a single UV-light source and two suitable emission filters for readout through a widely available BioImager device. For image and data processing, a customized software tool, the MultiFlow-Shiny app was used to accelerate and simplify the readout process. The app software provides advanced tools for image processing, including assisted extraction of line intensities, advanced background correction and an easy workflow for creation and handling of experimental data in quantitative LFAs. The results generated with our MultiFlow-Shiny app were superior to those generated with the popular software ImageJ and resulted in lower detection limits. Our assay is applicable for detecting clinically relevant ranges of both target proteins and therefore may serve as a powerful tool for POC diagnosis of inflammation and infectious events.
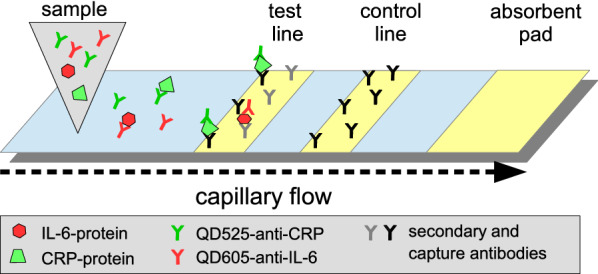

## Introduction

Sepsis, a life-threatening syndrome following a dysregulated host response to infection, frequently leads to organ dysfunction; it is a major public health concern because of its high mortality rates [1]. Because unspecific pathologies pose difficulties in diagnosis, the definition of sepsis has developed over time. The most recent international consensus on the definition of sepsis and septic shock, sepsis-3, was published in 2016 and defines diagnostic guidelines including hypotension, a decreased respiratory rate and a decrease in lactate levels. The *Quick Sequential Organ Failure Assessment* (qSOFA) score was further introduced for fast identification in patients at high risk [2]. Distinguishing sepsis from systemic inflammatory response syndrome (SIRS), which is not caused by a microbial insult, remains difficult, but this distinction is essential to determine proper treatment. For example, if a non-microbial event, such as trauma or necrosis, is the cause of inflammation, administration of antibiotics may cause unnecessary stress and increased mortality [3]. Sepsis leading to organ failure frequently involves the so-called cytokine storm, which also leads to complications in patients with COVID-19 [4]. Therefore, to achieve efficient therapeutic approaches, there is a major clinical need for biomarker assays with a fast turnaround time of ≤ 30 min to diagnose sepsis and guide therapy. Currently, no single biomarker can be used for the diagnosis of sepsis. However, evidence suggests that combined determination of multiple biomarkers might compensate for the low sensitivity and specificity of single marker molecules [5].

C-reactive protein (CRP), the clinically most important acute-phase protein, and interleukin-6 (IL-6) are both early biomarkers that can provide valuable information for distinguishing non-microbial SIRS from sepsis [6, 7]. The 120 kDa pentamer of CRP binds polysaccharides in pathogens and subsequently activates the complement pathway [3, 5, 6]. Under normal conditions, CRP levels are approximately 0.8 mg/L (38 nM) and do not exceed 10 mg/L (477 nM). Elevated CRP levels are indicative of an inflammatory process [6]; these levels can rise to up to 500 mg/L (24,000 nM) in severe cases. The proinflammatory cytokine IL-6 was chosen as the second target, because it is observed very early after noxious events and is produced almost instantly by B and T cells in response to bacterial pathogens. IL-6 weighs approximately 20 kDa, and normal levels are lower than 10 ng/L (0.5 pM). In noxious events, the IL-6 levels can rise as high as 1 µg/L (48 pM) [8]. Indeed, CRP and IL-6 levels both substantially differ between non-septic and septic patients, as well as between septic patients and patients with SIRS, thereby allowing for no sepsis, sepsis and SIRS to be differentiated [9, 10]. Furthermore, accurate quantification of CRP and IL-6 in sepsis and COVID-19 may be crucial for predicting outcomes, thus potentially enabling early therapeutic interventions and therapy control, e.g., in response to mechanical ventilation or Tocilizumab treatment [11–13]. Indeed, several other molecules, such as procalcitonin, are frequently described as potential biomarkers for sepsis [7, 14] and therefore may be included in further development of lateral flow assays, to increase the specificity of such point of care (POC) devices. A combination of CRP and IL-6 in POC devices would have numerous potential areas of application. For example, combined quantification of CRP and IL-6 might be useful for the detection of periprosthetic hip infections [15]. Furthermore, different concentration ranges for CRP, as well as for IL-6, have been shown to serve as risk indicators for coronary artery disease [16–18]. The relevant detection range of both CRP and IL-6 is, however, highly dependent on the intended application.

**Fig. 1:**
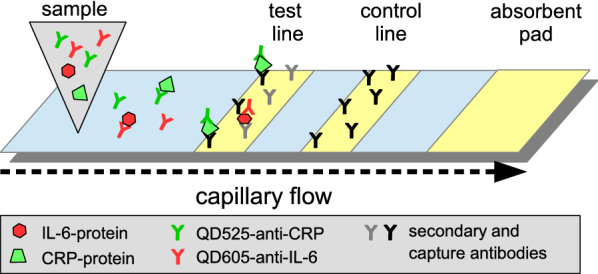
Illustration of a lateral flow sandwich immunoassay. Antibodies to CRP/IL-6 bound to the test line, and the control line with secondary antibody (anti-mouse/-goat)

Lateral flow immunoassays (LFIAs) are simple, rapid, robust and cost-effective devices with demonstrated potential to simplify and accelerate diagnostics in laboratory settings as well as in resource-poor environments; therefore, LFIAs are a preferable choice for POC diagnostics. Furthermore, the desired concentration ranges can easily be adjusted by varying the applied sample volumes or adding competing unlabeled antibodies, thus rendering LFIAs highly flexible. In addition, different design approaches can be used in lateral flow assays, such as sandwich assays (Fig. 1) or a competitive design to enable detection of small molecules [19, 20].

**Fig. 2:**
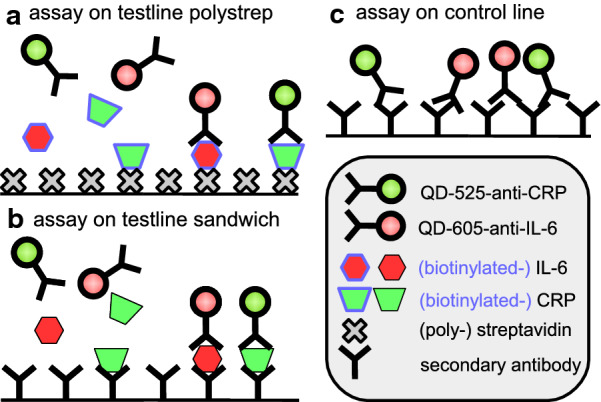
Binding reaction on the lateral flow strip. **a** Model system: CRP/IL-6-Biotin conjugates bind streptavidin bound to the test line. **b** Sandwich immunoassay: antibodies to CRP/IL-6 bound to the test line. **c** Control line reaction with secondary antibody (anti-mouse)

In competitive design, if antigen is present in the sample, bioprobes consisting of an antibody conjugated to a dye particle will be saturated and unable to bind the test line (see Fig. 2a). If no or little analyte is present in the sample, the bioprobe binds at the test line (see Fig. 2b). Saturated probes are captured at the control line by secondary antibodies, thus indicating that the test is valid [19, 20]. A positive assay shows one test line. The information obtained from each test line can be multiplied by using bioprobes tagged with distinct colors; accordingly, more distinct parameters can be investigated in one lateral flow assay (LFA). To date, mixing different colors at one test line has been achieved only with chromogenic bioprobes [21–23]. A mixture of fluorescent bioprobes has been used only with readout on separate test lines [24].

For detection, antibodies to CRP and IL-6 were conjugated to green and red emitting semiconductor nanocrystals, so-called quantum dots (QDs). QDs can be excited simultaneously by a single UV-light source, emit narrow, sharp peaks of a distinct color, are very resistant to photodegradation and have high fluorescence intensities, which makes QDs a very favorable and effective label for duplex or multiplex approaches in bioassays like LFAs [25, 26].

## Materials and methods

### Reagents

For synthesis of QD labeled antibody conjugates, we used amine modified CANdot SeriesA QDs (em. max. 530 and em. max. 610) purchased from CAN (Hamburg, Germany) and carboxyl modified QDs (Qdot 525 ITK, Qdot 605 ITK) from Thermo Fischer Scientific (Waltham, USA). Two anti-human IL-6 antibodies (polyclonal host: goat; monoclonal host: mouse), two anti-human-C-reactive protein antibodies (polyclonal host: rabbit; monoclonal host: mouse) and recombinant human IL-6 were obtained from Peprotech (Hamburg, Germany). Secondary anti-mouse (polyclonal host: goat; anti-heavy and light chain IgG, IgA and IgM) antibodies for generation of the control lines were purchased from Sigma Aldrich (St. Louis, USA). Buffers and reagents were purchased from Sigma Aldrich. Human CRP was purchased from Life Diagnostics (West Chester, USA). Biotinylated IL-6 and CRP were provided by R-Biopharm (Darmstadt, Germany). All buffers and reagents were prepared with milliQ water (≥ 18 MΩ). Pur-A-Lyzer Midi (10 kDa MWCO) dialysis tubes and Vivaspin 500 (15 kDa MWCO) columns were purchased from Sigma Aldrich (St. Louis, USA). Lateral flow test strips with a streptavidin test line and anti-mouse-antibody control line were provided by R-Biopharm. CN 95 and CN150 lateral flow membranes were obtained from Sartorius (Goettingen, Germany).

### Synthesis of QD labeled antibodies

Amine QD (CANdot-530-anti-CRP and CANdot-610-anti-IL-6) antibody conjugates were prepared with the following protocol.

For activation of QDs (CANdots Series A, amine) 1 µL stock solution (5 µM) was diluted in 184 µL 1× PBS (1 mM EDTA, pH 8.5), and 10 µL of SMCC (125 µM in milliQ water) was added. The mixture was incubated on a horizontal shaker at 22 °C for 1 h. The solution was then dialyzed (10 kDa MWCO) against 1× PBS (1 mM EDTA, pH 7) for 45 min to remove excess SMCC linker. After dialysis, the volume was adjusted to 250 µL with 1× PBS (1 mM EDTA, pH 8.5).

For antibody activation, 1 µL of antibody (anti IL-6 or CRP; 2.25 mg/L in milliQ water) was dissolved in 359 µL 1× PBS (1 mM EDTA, pH 7). Then 10 µL of Traut’s reagent (2-Iminothiolan, 16.5 µM, tenfold excess) was added to a final volume of 370 μL. The mixture was incubated for 1 h at 500 rpm and 22 °C. Excess Traut’s reagent was removed with a centrifugal concentrator (Vivaspin 500, 15 kDa MWCO), and the antibodies were washed twice with 500 µL PBS (1 mM EDTA, pH 7). Antibodies were then re-dispersed in 370 µL 1× PBS (1 mM EDTA) and combined with the activated QD-solution. The reaction mix was incubated for 30 min at 500 rpm and 22 °C. Then 50 µL 1% BSA in milliQ water was added to the solution to a final volume of 620 µL, and the conjugates were stored at 4 °C overnight.

Carboxyl QDs (Qdot-525-anti-CRP-conjugate and Qdot-605-anti-IL-6-conjugate) antibody conjugates were prepared with the following protocol.

A total of 5 µL of Qdot ITK stock solution (8 µM) was dissolved in 50 µL MES Buffer (50 mM, pH 6.4). Then 5 µL EDC (10 mg/mL in milliQ water) and 5 µL sulfo-NHS (10 mg/L in milliQ water) were added; the mixture was incubated for 30 min at 500 rpm and 22 °C. Then 135 µL of MES buffer (pH 6.4) and 80 µL of antibody solution (0.5 µg/mL in PBS, pH 7.4) were added to a final volume of 200 µL; the mixture was incubated for 90 min at 500 rpm and 22 °C. Then 150 µL HEPES buffer (50 mM, 0.1% Tween 20 and 10% BSA, pH 7.4) was added to a final volume of 350 µL, and the conjugates were stored at 4 °C overnight.

QD (carboxylated) conjugates were characterized by fluorescence emission spectra, agarose gel electrophoresis and dynamic light scattering to verify successful conjugation (Additional file 1: Section S1.1). Fluorescence spectra measurements were collected with a TECAN infinite 200Pro plate reader from Tecan Group Ltd. (Männedorf, Switzerland). Briefly, the prepared conjugates were diluted in ddH_2_O to 100 µL. Afterward, QDs were excited at 365 nm, and the fluorescence emission between 450 and 600 nm for QD525, or 550 and 700 nm for QD605, was recorded. Emission peaks were normalized to the peak maximum by dividing the emission values by the maximum emission value. Agarose gel electrophoresis of QDs before and after conjugation to the corresponding antibodies was performed with 0.5% (w/v) agarose gel electrophoresis in 1× Tris-acetate-EDTA buffer. Electrophoresis was performed at 10 V/cm for 20 min, and pictures were taken with a Gel iX20 Imager device (Intas, Göttingen, Germany). Dynamic light scattering measurements were performed with a Zetasizer Nano instrument (Malvern, Worcestershire UK).

### Test strip production and LFA assay procedure

Three different systems were used in the development of the duplex LFA for detection of CRP and IL-6 through optical duplex detection:

1. Streptavidin assay strip production
  Streptavidin assays were performed on lateral flow strips with polystreptavidin on the test line and anti-mouse secondary antibodies on the control line (provided by R-Biopharm). Biotin labeled CRP and IL-6 proteins were used as analytes. For detection of bound biotinylated proteins, QD labeled (CAN dots Series A, 530/610 em. max.) antibody conjugates, CANdot-530-anti-CRP and CANdot-610-anti-IL-6 were applied.
2. Sandwich assay (0–20 nM) and clinical range assay strip production
  The sandwich assay LFA-strips were produced by printing anti-CRP and anti-IL-6-antibodies (0.5 mg/mL anti-CRP polyclonal rabbit; 0.5 mg/mL anti-IL-6 polyclonal rabbit) on the test line and secondary antibodies (1 mg/mL anti-mouse polyclonal goat) on the control line, at a density of 4.85 µL/cm for each line, by using a lateral flow reagent dispenser (Claremont BioSolutions, USA). For the sandwich assay, Sartorius CN95 fast wicking lateral flow membrane was used. For the clinical range assay, Sartorius CN150 high sensitivity lateral flow membrane was used. After printing, the lateral flow membranes were dried overnight in a desiccator at room temperature. The membranes were then affixed to absorbent filters (Whatman) with adhesive tape and cut into 5 mm-wide LFA-strips.
3. Assay procedure
  For all lateral flow tests, running buffer (Bis-Tris 50 mM, 8% Triton X-100 and 0.3% BSA, pH 6.4) was used. Sample proteins were dissolved in 1× PBS with a content of 0% (streptavidin assay), 1% (sandwich assay) or 10% (clinical range assay) human serum. For the streptavidin assay, biotin-labeled target proteins were used. The volume for one lateral flow test sample preparation was 100 μL or 120 µL, consisting of 5–10 µL QD-conjugates of each color, 10 µL or 50 µL target protein solution, and 55 or 70 µL running buffer. The sample mixture was prepared in 2 mL flat bottomed reaction vessels and incubated for 1 min. Then test strips were placed upright in the prepared vessels for either 10 or 20 min to allow the sample mixture to flow through the membranes. After the run, the test strips were placed on a benchtop to dry for 10 min and then imaged. Sample preparations for different assays are summarized in Table 1.

**Table 1:** Sample preparation and processing of LFAs

	Streptavidin assay	Sandwich assay 0–20 nM	Clinical range assay
QD-antibody conjugate	10 µL CANdot-530-anti-CRP; 10 µL CANdot-610-anti-IL-6, both undiluted	10 µL Qdot-525-anti-CRP; 10 µL Qdot-605-anti-IL-6, both undiluted	10 µL Qdot-525-anti-CRP, diluted 1:1 with 0.3 µg/mL anti-CRP (mouse); 5 µL Qdot-605-anti-IL-6, undiluted
LFA-test strips	Test line, polystreptavidin; control line, anti-mouse secondary antibody	Test line, anti CRP (rabbit)/anti IL-6 (goat); control line, anti-mouse- secondary antibody; membrane CN95	Test line, anti CRP (rabbit)/anti IL-6 (goat); control line, anti-mouse- secondary antibody; membrane CN150
Sample composition	20 µL QD-conjugate 10 µL CRP/IL-6 (biotinylated), 0–20 nM in 1× PBS (pH 7.4) 70 µL running buffer	20 µL QD conjugate 10 µL CRP/IL-6, 0–20 nM in 1× PBS (1% serum, pH 7.4) 70 µL running buffer	15 µL QD conjugate 50 µL CRP (0–1000 nM)/IL-6, (0–60 pM) in 1× PBS (10% serum, pH 7.4) 55 µL running buffer
Assay time	1 min incubation of sample mix 10 min run time 10 min drying Imaging ≤ 2 min	1 min incubation of sample mix 10 min run time 10 min drying Imaging ≤ 2 min	1 min incubation of sample mix 20 min run time 10 min drying Imaging ≤ 2 min

### Imaging procedure

Images of test strips were acquired with a BioImager (ChemStudio PLUS, Analytik Jena) equipped with the following bandpass emission filters: filter 1, green channel, 513–557 nm (used with CANdot-530-anti-CRP and Qdot-525-anti-CRP); filter 2, red channel, 565–625 nm (used with CANdot-610-anti-IL-6 and Qdot-605-anti-IL6). Illumination/excitation of fluorescent QD-conjugates was performed with an inbuilt UV-light (top light, λ = 365 nm). Two pictures were taken with each emission filter at 16 MP resolution (highest resolution, for streptavidin and sandwich assays) or 2 × 2 binning (for clinical range assays). Depending on the experimental setup, as well as the analyzed QD, illumination times between 1 and 20 s were chosen to achieve images with clearly visible test and control lines but no oversaturation of the lines, which has been demonstrated to have a negative influence on the readout of AUC values in ImageJ or the developed MultiFlow-Shiny app, thus leading to flat readout peaks (oversaturated peaks). Illumination times were kept constant for each experimental setup and the corresponding QD conjugates used. A detailed list of the imager settings used is shown in Additional file 1: Section S1.2.

### MultiFlow-Shiny app

For image processing and data collection, we programmed readout software based on several packages of R statistical software for analysis of bioassay data, which was implemented in our MultiFlow-Shiny app [27–30]. We processed all acquired image datasets with our app and with the ImageJ (V1.50i) gel analyzer tool and compared the acquired key measures such as limit of blank (LOB), limit of detection (LOD) and limit of quantification (LOQ) [31, 32].

Our data processing MultiFlow-Shiny app can be used to process colorimetric data lists from AUC values acquired with other software, such as ImageJ, or can be used as an all-in-one solution for image readout with automated generation of a results sheet. The software allows for cropping, segmentation and background correction of the images to generate the background corrected intensity values for the bands. It combines the intensity data with experimental data, can average technical replicates and computes linear calibration curves. Furthermore, an .html report is generated, including full details about the calibration analysis. The development version of our R packages including the MultiFlow-Shiny app can be downloaded from https://github.com/stamats/MultiFlow. Further details on the MultiFlow app and an illustrated users guide for the use is available from https://stamats.github.io/MultiFlow/MultiFlow.html; a video tutorial is available from https://www.youtube.com/playlist?list=PLRgOZXM8LZ0gv2OJts1c62n0gsXO9VrAN. In the app, imported image files of LFA-strips were first cropped and segmented to select the area of interest and to include a visual control with the test and control lines being properly positioned to enable subsequent background subtraction through Otus’s method and readout of line intensity values [33]. The acquired values were merged with the experimental information and exported as a .csv file, which was used to calculate concentration values derived from the duplex CRP/IL-6 LFA. The app allowed us to create custom calibration profiles for strip based bioassays and generate .html reports with the results of the calibration analysis as well as the key measures LOB, LOD and LOQ.

## Results and discussion

### Streptavidin assay

The objective of the experiments was to evaluate whether two analytical targets could be quantitatively detected at the same test line by using two different fluorescent labels. Therefore, biotinylated analytes (biotinylated CRP and biotinylated IL-6) in combination with two different QD-antibody conjugates (CANdot-530-anti-CRP and CANdot-610-anti-IL-6) were used. After binding of biotinylated targets to the corresponding antibody-QD-conjugates, the complex was bound on the streptavidin test line (Fig. 2a). Conjugates without target did not bind the test line but were captured on the control line containing secondary antibodies.

We first intended to use the system as a competitive immunoassay, in which the added target proteins, CRP/IL-6 without a biotin label, would compete for antibody binding, thus decreasing the fluorescence signal intensity on the test line with increasing target concentration. However, the competitive assay did not show a quantitative correlation after evaluation of the data obtained via ImageJ (Additional file 1: S2.1). We assume that this result was due to high amounts of target competing for the antibody as well as biotin/streptavidin binding sites. Indeed, evaluation with our own data processing MultiFlow-Shiny app revealed a concentration dependent decrease in test line signal intensity (Additional file 1: S2.1). However, the variability and linearity remained poor, as indicated by the low coefficient of determination. Therefore, we switched to a sandwich immunoassay approach, decreasing the number of required components.

### Sandwich LFA

**Fig. 3:**
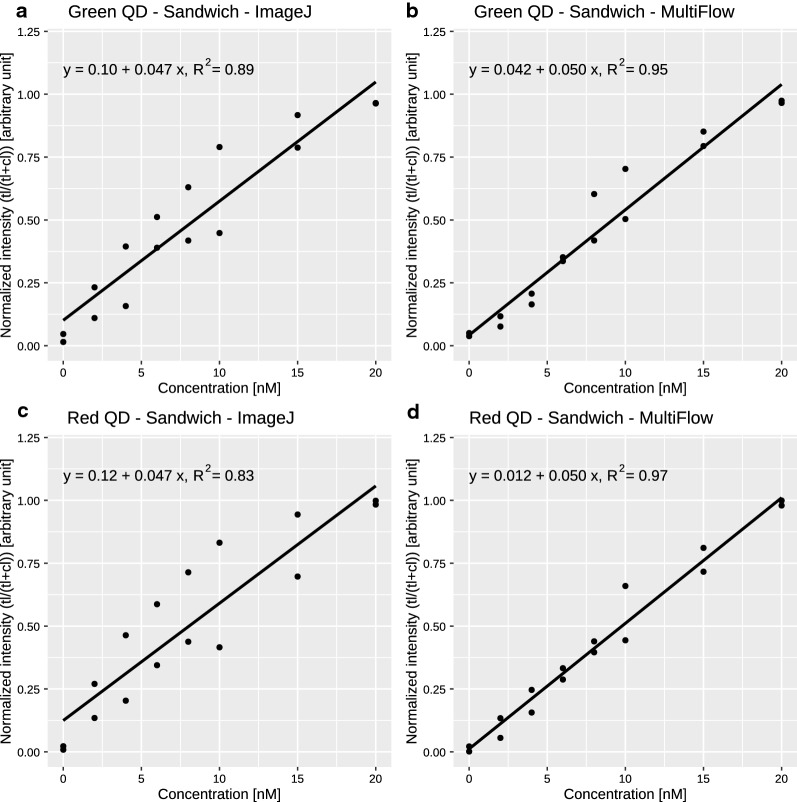
Calibration curves of the LFA sandwich assay (0–20 nM)

After demonstrating that the system generated quantitative data in the streptavidin assay, whereas the competitive assay format was unsuccessful, we designed a new LFA setup based on a sandwich immunoassay format. The test line was composed of anti-CRP and anti-IL-6 antibodies, and QD-525-anti-CRP and QD-605-anti-IL-6 antibodies were used with unlabeled CRP and IL-6 proteins as targets. Initially, we decided to use similar concentration ranges for both analytes to evaluate the linearity at comparable intensities. Because the relevant concentration ranges for both analytes differed by several orders of magnitude, we initially decided to use concentrations between 0 and 20 nM for both analytes. Indeed, when we used the data obtained from ImageJ as well as from our MultiFlow-Shiny app, the sandwich immunoassay format clearly showed a concentration dependent signal increase for both analytes in the range of 0–20 nM. Nevertheless, the variability in the intensities obtained from ImageJ analysis still remained poor. Additional analysis with our MultiFlow-Shiny app, however, showed significantly lower variability and enhanced the limit of detection and of quantification (Fig. 3 and Table 2). This result was probably due to the automated intensity measurement in combination with background correction in the MultiFlow-Shiny app; in contrast, in classical ImageJ analysis, these parameters are defined by the user.

**Table 2:** Key measures for the sandwich LFA

Sandwich assay 0–20 nM	Green channel QD-525-anti-CRP		Red channel QD-605-anti-IL-6	
Image processing software	ImageJ	MultiFlow app	ImageJ	MultiFlow app
LOB	Negative	0.33	Negative	0.46
LOD	2.28	1.27	1.38	2.28
LOQ	11.12	5.52	9.49	7.33
R^2^ of linear fit	0.89	0.95	0.83	0.97

Figure 3 shows the calibration curves for the range of 0–20 nmol/L for CRP and IL-6. The R^2^-values were clearly better for data acquired with the MultiFlow-Shiny app (0.95 and 0.97) than with *ImageJ* (0.89 and 0.83). Overall, data processing through the MultiFlow-Shiny app provides a benefit over *ImageJ*, a popular, widely used standard tool for quantification of laboratory data. The MultiFlow-Shiny app is a user-friendly solution for readout and data processing of functional LFAs that can be used not only for QD labeled antibodies but also for any kind of LFA, in principle containing an arbitrary number of bands with one or more color labeled antibodies.

### Clinical range assay

**Fig. 4:**
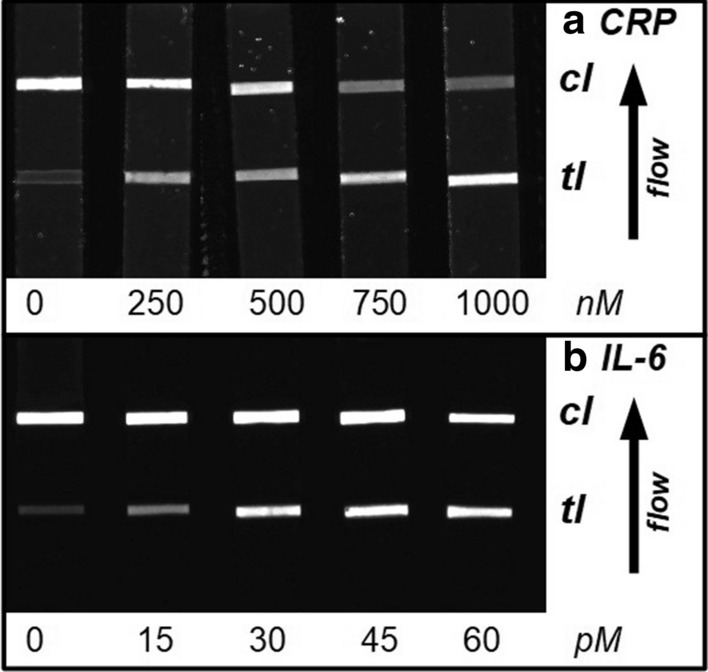
CRP/IL-6 duplex LFA strips (0–1000 nM CRP/0–60 pM IL-6); top, CRP-assay (readout 513–557 nm bandpass filter); bottom, IL-6-assay (readout 565–625 nm bandpass filter); *cl* control line, *tl* test line. Brightness and contrast were adjusted for better visibility of lines

**Fig. 5:**
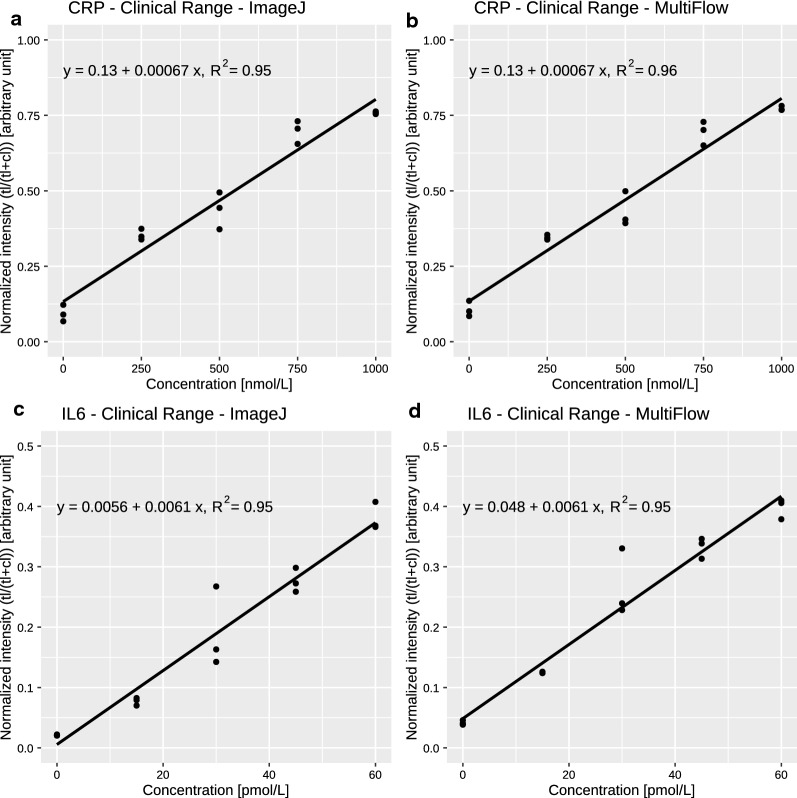
Calibration curves of the clinical range LFA assay: CRP range, 0–1000 nM; IL-6 range, 0–60 pM

As described in “Introduction”, the relevant clinical range of CRP is between 38 and 24000 nM, whereas the clinical range of IL-6 is much lower, between 0.5 and 48 pM. Because our initial sandwich immunoassay was developed by using a range between 0 and 20 nM, the assay needed to be adjusted to better reflect the relevant concentration ranges observed during inflammatory events such as sepsis or bacterial/viral infections. Therefore, the detection limit of IL-6 was decreased to below 48 pM through increasing the sample amount used per LFA from 10 to 50 µL; using a slow wicking, high sensitivity lateral flow membrane; and decreasing the amount of Qdot-605-anti-IL-6-conjugate to decrease the background fluorescence. To compensate for low emission, we decreased the resolution of the CCD-camera from a maximum of 16 MP resolution to 2 × 2 binning settings, thus allowing for a fast acquisition time of 1 s while maintaining the brightness of the test lines to be detected. The CRP concentration in blood samples is of interest if it exceeds 500 nM; therefore, the detectable concentration needed to be adjusted to accommodate higher amounts. This was achieved by dilution of the Qdot-525-anti-CRP conjugates with additional anti-CRP antibodies, which competed with the QD-conjugates for the target protein (the sample composition of all three assay types can be found in Table 1). Using these simple modifications, we were able to adjust the CRP/IL-6 assay to the clinically relevant range (examples of test strips in Fig. 4; linear calibration models of both analytes in Fig. 5 and key measures in Table 3), thus enabling the immediate applicability of our assay.

**Table 3:** Key measures for the clinical range LFA

Clinical range assay	Green channel QD-525-anti-CRP (nM)		Red channel QD-605-anti-IL-6 (pM)	
Image processing software	ImageJ	MultiFlow app	ImageJ	MultiFlow app
LOB	7.9	22.8	2.8	Negative
LOD	52.9	42.5	4.5	0.21
LOQ	556.4	527.7	15.3	16.4
R^2^ of linear fit	0.96	0.95	0.95	0.95

### Performance of the MultiFlow-Shiny app

**Fig. 6:**
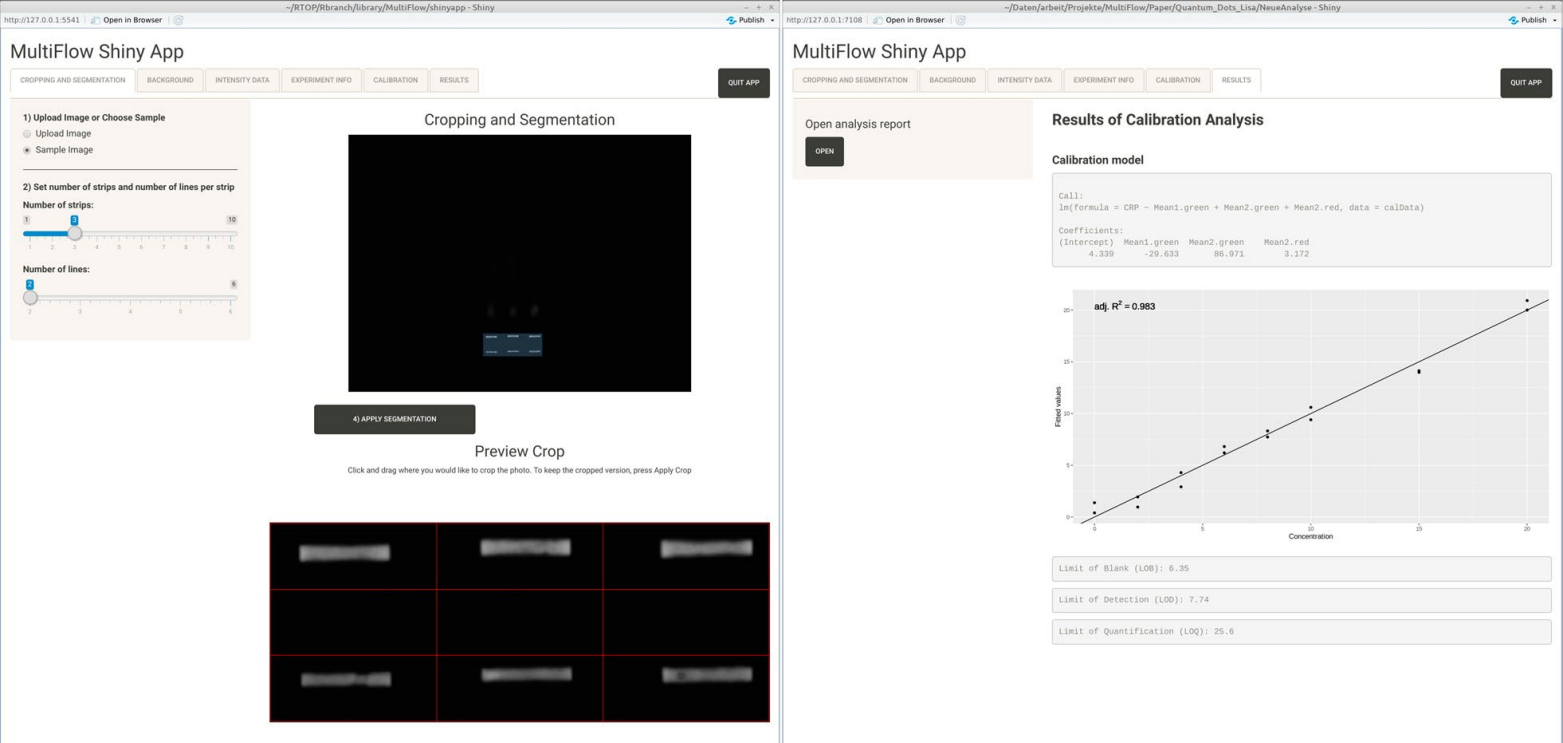
Screenshot of the MultiFlow-Shiny app: left, cropping and segmentation; right, result report generation

The MultiFlow-Shiny app provides an all in one solution for the analysis of images taken from LFAs that may include up to six lines, a restriction we chose since we are not aware of any LFA having more than six lines. It works for grayscale as well as color images and can handle images that include several well aligned strips in one batch. Overall, it clearly speeds up the analysis process compared to other image analysis software such as ImageJ. It provides various tools for processing the images, handling the intensity and the experimental data, conducting a calibration analysis by arbitrary linear models and generating automatic.html reports of the calibration analysis. Furthermore, the app offers various options to start the analysis. Instead of starting with the raw images, one can also start with already existing intensity data (e.g. from ImageJ) or further preprocessed intensity data (e.g. after averaging technical replicates). The results of the MultiFlow-Shiny app are also well reproducible, since the analysis is fully automatic except for the cropping of the images. We found that the app especially outperforms the manual analysis with ImageJ when the analyzed LFAs contain very weak signal intensities or broad and blurred lines and consequently leads to better calibration with higher measures of determination. Figure 6 shows screenshots of the user interface of the MultiFlow-Shiny app.

## Conclusion

The presented LFAs were designed to detect the sepsis biomarkers CRP and IL-6 simultaneously on one test line, by using two different QDs as labels. We calibrated the LFAs (streptavidin, sandwich assay and clinical range assay) by using linear models, and we demonstrated that optical duplex imaging using emission filters for signal separation did not indicate any mutual disturbance between different QD-dyed antibody probes. The results therefore indicated that the presented setup is suitable for quantitative readout. Data processing with our MultiFlow-Shiny app with automated report generation significantly increased the test performance relative to that of a general-purpose standard software solution, such as *ImageJ*. This improvement was particularly evident in the detection of LFA lines with very weak signal intensities or wide and blurred lines. Accordingly, we not only achieved but exceeded the sensitivity required for the detection of CRP in clinical diagnostics. We furthermore demonstrated that, with simple adjustments (e.g., varying the sample volume, amount of probes applied, addition of unlabeled antibodies and different lateral flow membranes), this method can be made suitable for detecting clinically relevant concentration ranges, thus providing a highly useful POC assay. Similar approaches should be feasible for other targets. The setup presented, with its optimization to clinical parameters, has potential for increased number of analytical targets and optimized readout workflow through our app. Together with the downsizing of readout equipment, the assay has promise as a robust, inexpensive and rapid POC sensing system for sepsis and other diagnostic challenges.

## Supplementary information

**Additional file 1.** Supplementing information of material characterization, imaging hardware settings and results of data processing for the streptavidin and clinical range assay.

## Data Availability

MultiFlow-Shiny app download: https://github.com/stamats/MultiFlow Manual at: https://stamats.github.io/MultiFlow/MultiFlow.html Video tutorial: https://www.youtube.com/playlist?list=PLRgOZXM8LZ0gv2OJts1c62n0gsXO9VrAN Additional charts and tables referred to in the text are available in Additional file 1.

## Abbreviations

CRP: C-reactive protein
IL-6: Interleukin-6
LF(I)A: Lateral flow (immune) assay
QD: Quantum dot
LOD: Limit of detection
LOB: Limit of blank
LOQ: Limit of quantification
POC: Point of care
PBS: Phosphate buffered saline
EDTA: Ethylenediaminetetraacetic acid
SMCC: *N*-Succinimidyl 4-(maleimidomethyl)cyclohexanecarboxylate
BSA: Bovine serum albumin
MES: 2-(*N*-Morpholino)ethanesulfonic acid
EDC: *N*-Ethyl-*N*′-(3-dimethylaminopropyl)carbodiimide
sulfo-NHS: *N*-Hydroxysulfosuccinimide
HEPES: 4-(2-Hydroxyethyl)piperazine-1-ethanesulfonic acid
Tween 20: Polyethylene glycol sorbitan monolaurate
Bis-Tris: 2,2-Bis(hydroxymethyl)-2,2′,2″-nitrilotriethanol
Triton X-100: *t*-Octylphenoxypolyethoxyethanol
