# Supplementary material for: Duplex Shiny app quantification of the sepsis biomarkers C-reactive protein and interleukin-6 in a fast quantum dot labeled lateral flow assay
Source: J Nanobiotechnology. 2020 Sep 10;18:130. doi: 10.1186/s12951-020-00688-1 (PMC7481553; doi:10.1186/s12951-020-00688-1)
Supplement: Supplementary file 1 — Additional file 1. Supplementing information of material characterization, imaging hardware settings and results of data processing for the streptavidin and clinical range assay. [file 12951_2020_688_MOESM1_ESM.docx]

**Additional Material on the Journal of Nanobiotechnology publication entitled:**

**Duplex Shiny app quantification of the sepsis biomarkers C-reactive protein and interleukin-6 in a fast quantum dot labeled lateral flow assay**

**Authors:** Christoph Ruppert, Lars Kaiser, Lisa Johanna Jacob, Stefan Laufer, Matthias Kohl and Hans-Peter Deigner

**S1 Materials and methods: p.2**

**S1.1 Material characterization p.2**

**S1.2. Imaging Hardware-Settings p.3**

**S2 Results of data processing p.4**

**S2.1 Strepavidin assay p.4**

**S2.2 Clinical range assay p.5**

**S1 Materials and methods:**

**S1.1. Material characterization**


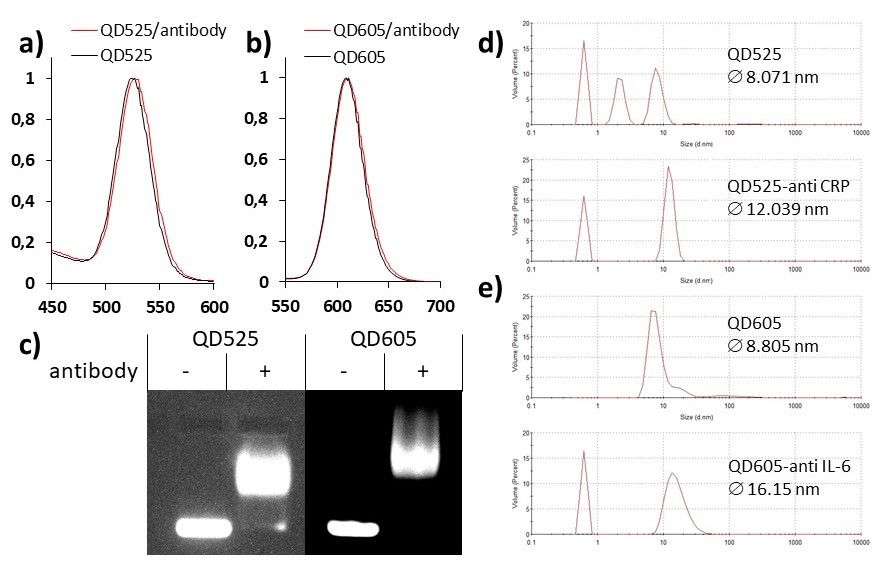


Figure S1.1: Characterization of prepared Quantum Dot – antibody conjugates. (a,b) Fluorescence emission spectra of QD525 (a) and QD605 (b), prior (black curve) and after (red curve) antibody conjugation.(c) Agarose gel electrophoresis of Quantum Dots prior (-) and after (+) conjugation to antibodies against CRP (QD525) or IL-6 (QD605). (d,e) DLS plots of QD525 (d) and QD602 (e) prior (upper chart) and after (lower chart) conjugation with antibody.

**S1.2. Imaging Hardware-Settings**

For image acquisition a *ChemStudio Plus* BioImager (Analytic Jena) equipped with a 16 Megapixel CCD-camera was used. For all quantum dot dyes the inbuild UV toplight source at λ=365nm center wavelength was used.

Green Channel: For readout of CANdot-530-anti-CRP / Qdot-525-anti-CRP

Emission filter: 513-557 bandpass filter (Omega Optical nr. 535AF45)

Illumination time: Streptavidin Assay/Sandwich Assay: 20 s 16MP resolution

Clinical Range Assay: 4s 2x2 binning

Green Channel: For readout of CANdot-610-anti-IL-6 / Qdot-605-anti-IL-6

Emission filter: 565-625 bandpass filter (Omega Optical nr. 595BP60/50SQ)

Illumination time: Streptavidin Assay/Sandwich Assay: 4 s 16MP resolution

Clinical Range Assay: 1s 2x2 binning

Images were exported as *.tiff* file and further processed either through *ImageJ* (V. 1.50i) or *the Multiflow-Shiny app*

**S2 Results of data processing**

**S2.1 Streptavidin assay**

Fig. S2.1: Calibration curves of the Streptavidin, range 0-20nmol/L

**S2.2 Clinical range assay**

Fig. S2.2: Calibration curves of clinical range assay for CRP singleplex

Fig. S2.3: Calibration curves of clinical range assay for IL-6singleplex
